# Change in the perioperative blood glucose and blood lactate levels of non-diabetic patients undergoing coronary bypass surgery

**DOI:** 10.3892/etm.2013.1268

**Published:** 2013-08-22

**Authors:** CHUNJIAN SHEN, TIANXIANG GU, LILI GU, ZHONGYI XIU, ZHIWEI ZHANG, ENYI SHI, YUHAI ZHANG, CHUN WANG

**Affiliations:** 1 Department of Cardiac Surgery, The First Hospital of China Medical University, Shenyang, Liaoning 110001;; 2 China Medical University, Shenyang, Liaoning 110001;; 3 Shenzhou Hospital of Shenyang Medical College, Shenyang, Liaoning 110002, P.R. China

**Keywords:** coronary artery bypass graft, cardiopulmonary bypass, blood glucose, blood lactate

## Abstract

In the present study, a retrospective analysis of the trends and factors affecting blood glucose and blood lactate levels was carried out for non-diabetic adult patients who had undergone coronary artery bypass graft (CABG) surgery. Between October 2009 and October 2011, 200 non-diabetic adult patients undergoing CABG surgery were examined. Glucose and lactic acid levels were observed successively during surgery [following the induction of anesthesia, cardio-pulmonary bypass (CPB), aortic cross-clamping and aortic stop flow infusion], at the end of surgery and after surgery [1, 6, 12, 24 and 48 h after admission to the intensive care unit (ICU)]. The results of blood gas analyses and other related indicators for trend analysis were investigated. The peak blood glucose and blood lactate levels during CPB, as well as other CPB factors, were also analyzed. Following aortic cross-clamping, intraoperative blood glucose and blood lactate levels increased gradually with increasing operative time. Postoperatively, blood glucose and blood lactate levels continued to rise. Blood glucose and blood lactic acid levels during CPB were positively correlated. The blood glucose and blood lactate levels of non-diabetic adult patients undergoing CABG increased gradually with operative time following aortic cross-clamping. Moreover, blood glucose and blood lactate levels were positively correlated with the duration of CPB and duration of aortic cross-clamping.

## Introduction

During extracorporeal circulation in cardiac surgery, also known as cardiopulmonary bypass (CPB), the internal environment undergoes significant changes due to anesthesia, CPB, surgical operation and other factors. The phenomenon of increased blood glucose and blood lactate levels during the perioperative period is often observed, even in patients with hyperglycemia (1–5) 60–70% (4) and lactic acidosis (6–8) 10–20% (9,10). This increases the incidence of postoperative complications and mortality (1,8,9,11). At present, patients with diabetes, particularly those who have previously undergone CPB or coronary artery bypass graft (CABG) surgery of the downstream arteries, more frequently experience and report blood glucose level changes. Treatment strategies have been developed for these patients. Reports of changes in blood glucose and lactate levels and the need for intervention in non-diabetic patients are rare (4). In the present investigation, a dynamic study of the trends of blood glucose and lactate levels in non-diabetic adult patients undergoing CABG surgery was carried out.

This study was conducted in order to explore the prevention and treatment of high levels of blood glucose and lactic acidosis, in an attempt to improve CPB management, maintain the internal environment during surgery, reduce the incidence of CPB-related complications and improve prognosis.

During cardiac surgery, a continued increase of perioperative blood glucose levels to >200 mg/dl is considered to be hyperglycemia (12–16). High blood glucose increases the risk of postoperative mortality (1–5), postoperative infections (3,17), myocardial injury (18–20), stroke (2,21) and neurological dysfunction (2,22). A number of perioperative glycemic control standards have been established; however, reports concerning their clinical effectiveness are inconsistent (23,24). Ruesch *et al* (16) suggested that intraoperative blood glucose levels of >200 mg/dl require treatment (16). Lazar *et al* (14) reported that the perioperative management of blood glucose significantly improved prognosis in diabetic patients (n=141) with blood glucose levels of 125–200 mg/dl who underwent cardiac surgery. Based on 1,548 cases, Van den Berghe *et al* (25,26) suggested that blood glucose levels that reach 80–110 mg/dl after surgery should be controlled. Carr *et al* (27) reported that the perioperative blood glucose levels of patients undergoing cardiac surgery should be reduced to 110 mg/dl. Lazar (28) and Quinn *et al* (29) also emphasized the importance of postoperative management of blood glucose levels in non-diabetic and diabetic patients who undergo cardiac surgery. A blood lactate concentration of >3.0 mmol/l during the perioperative period of cardiac surgery is considered to be lactic acidosis (7,8). Whether lactic acidosis during CPB increases postoperative mortality remains controversial. Demers *et al* (9) reported that a peak blood lactate level of ≥4.0 mmol/l correlates with postoperative complications and mortality, based on a study of 1,376 cases. However, Ranucci *et al* (6) reported that lactic acidosis only increases the incidence of postoperative complications. Maillet *et al* (8) showed that blood lactate concentrations >3.0 mmol/l correlates with poor prognosis and an increased death rate following intensive care unit (ICU) admission.

In the present study, 200 non-diabetic patients undergoing CABG surgery were analyzed in order to explore treatment strategies for high blood glucose levels and lactic acidosis, and thus improve CPB management and prognosis.

## Materials and methods

### Patients

A total of 200 non-diabetic adult patients undergoing CABG surgery in Shenzhou Hospital (Shenyang, China) between October 2009 and October 2011 were included in this study. This study was conducted in accordance with the Declaration of Helsinki and with approval from the Ethics Committee of Shenzhou Hospital (Shenyang, China). Written informed consent was obtained from all participants.

Inclusion criteria were as follows: i) non-diabetic patients undergoing CABG surgery; ii) 31–65 years old (mean, 51±9 years old); iii) cardiac function, class II–III [New York Heart Association (NYHA) functional classification]; and iv) an ejection fraction (EF)>35%.

Exclusion criteria were as follows: i) diabetic patients, individuals exhibiting preoperative symptoms of diabetes and with random blood glucose levels ≥11.1 mmol/l (200 mg/dl) or fasting blood glucose levels ≥7.0 mmol/l (126 mg/dl); ii) liver or kidney dysfunction, abnormal liver function (alanine aminotransferase >40 U/l) or renal insufficiency [blood urea nitrogen (BUN) >16.0 mmol/l or creatinine (Cr) >170 *μ*mol/l]; iii) second surgery; iv) acute myocardial infarction and acute endocarditis; and v) cachexia with an intra-aortic balloon pump (counterpulsation) or left ventricular assist device.

### Data extraction

Blood glucose and blood lactate levels were obtained successively during surgery [after the induction of anesthesia, CPB, aortic cross-clamping (5–10 min) and aortic stop flow infusion (10 min after CPB, lasting for 10 min)], at the end of surgery and after surgery (1, 6, 12, 24 and 48 h following admission to the ICU). Results of blood gas analyses and other related indicators were recorded for trend analysis.

### Statistical analysis

All data were statistically analyzed using SPSS 11.5 (SPSS, Inc., Chicago, IL, USA). Measured data were expressed as the mean ± SD and compared using Student’s t-test and Spearman’s correlation analysis. P<0.05 was considered to indicate a statistically significant difference.

## Results

### Changes in perioperative blood glucose levels

During surgery, after aortic cross-clamping, blood glucose levels increased gradually with increasing operative time. The arterial (aorta) blood glucose levels significantly increased after the start of the operation and blood glucose levels peaked at the end of surgery.

**Figure 1.**
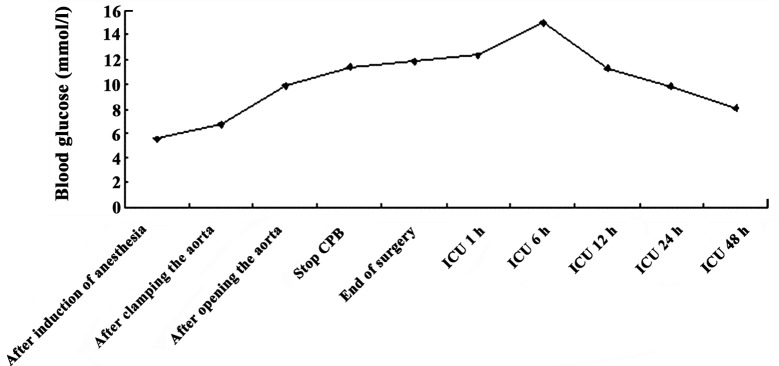
Intraoperative and postoperative trends in blood glucose levels following CABG in 200 adult non-diabetic patients. CABG, coronary artery bypass graft; CPB, cardiopulmonary bypass; ICU, intensive care unit.

After surgery, blood glucose levels continued to rise, and 6 h following admission to the ICU, they reached a peak of 15.01±4.91 mmol/l. After 12 h, the blood glucose levels began to decline and after 24 h, they returned to near-normal levels. However, a further decline (to 8.05±2.32 mmol/l) was observed after 48 h (Tables I and II; Fig. 1).

### Changes in perioperative blood lactate levels

During surgery, after aortic cross-clamping, blood lactate levels increased gradually with increasing surgery time. The mean arterial (aorta) blood lactate level was >4.88 mmol/l and lactic acidosis was observed. Following aortic stop flow infusion and CPB, blood lactic acid levels continued to rise significantly in a linear manner. The peak intraoperative blood lactate level occurred at the end of surgery.

**Figure 2.**
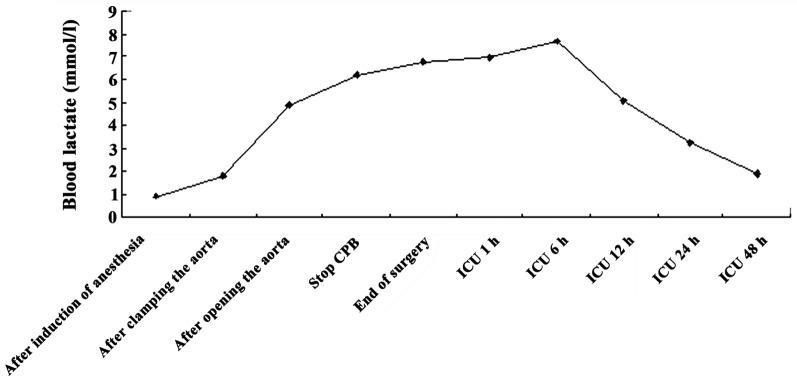
Intraoperative and postoperative trends in blood lactate levels following CABG surgery in 200 adult non-diabetic patients. CABG, coronary artery bypass graft; CPB, cardiopulmonary bypass; ICU, intensive care unit.

After surgery, blood lactate levels continued to rise, and 6 h following admission to the ICU, they reached a peak of 7.66±2.33 mmol/l. Thereafter, the blood lactate levels began to decline and returned to normal levels after 24 h. After 48 h, the blood lactate level was <2.0 mmol/l (Tables I and II; Fig. 2).

### Correlation analysis

As shown in Tables III and IV, the peak blood glucose and blood lactate levels during CPB were positively correlated (r=0.312, P=0.009). Peak blood glucose levels were positively correlated with the duration of the CPB procedure and aortic cross-clamping (r=0.386, P=0.001 and r=0.412, P<0.001, respectively). Peak blood lactate levels were also positively correlated with the duration of the CPB procedure and duration of aortic cross-clamping (r=0.489, P<0.001 and r=0.467, P<0.001, respectively).

Blood lactate levels increased with increasing blood glucose levels. During CPB, blood glucose and blood lactate levels exhibited increasing trends that correlated with the duration of CPB and aortic cross-clamping.

## Discussion

Lazar *et al* (14) observed that the intraoperative blood glucose levels of diabetic patients undergoing cardiac surgery increase gradually with increasing operative time when blood glucose levels are not controlled, and 6–12 h after CPB these levels reach a peak of >250 mg/dl (13.9 mmol/l). Therefore, during the perioperative period, insulin should be used to maintain blood glucose levels at <200 mg/dl (11.1 mmol/l). Prasad *et al* (4) reported that 70% of non-diabetic patients are often observed to have blood glucose levels >200 mg/dl (11.1 mmol/l) during CPB and 61% are considered to have high blood glucose. Of those who acquire postoperative hyperglycemia, 22% continue to have the condition. During cardiac surgery, perioperative blood glucose levels >200 mg/dl (11.1 mmol/l) are considered to be hyperglycemic. The incidence of intraoperative hyperglycemia is comparable to that of postoperative infection (3,17), myocardial injury (18–20), postoperative neurological injury, cognitive dysfunction and other complications (2,22) that result in adverse prognosis.

In the present study, intraoperative blood glucose levels determined during aortic cross-clamping at the aorta gradually increased with increasing operative time, reaching levels of 9.92±3.03 mmol/l. Following aortic stop flow infusion and CPB, blood glucose increased to >11.1 mmol/l. High blood glucose levels were observed at the end of surgery, and intraoperative blood glucose levels reached a peak of 11.83±4.32 mmol/l. Correlation analyses revealed that during CPB, peak blood glucose levels and the duration of aortic cross-clamping were positively correlated. These observations indicate that blood glucose levels of non-diabetic adult patients undergoing heart valve surgery gradually increase with increasing operative time and that hyperglycemia occurs during surgery. This suggests that the prevention and treatment of hyperglycemia is required during cardiac surgery.

Following surgery, blood glucose levels continued to rise and reached a peak of 15.01±4.91 mmol/l after 6 h in the ICU. Blood glucose levels decreased significantly to nearly 11.33 mmol/l after 12 h, returned to near-normal levels at 24 h and decreased to 8.05±2.32 mmol/l at 48 h. These observations indicate that an intervention for high blood glucose is necessary.

Lazar *et al* (14) observed that blood lactic acid levels increase with increasing operative time during CPB, reach peak levels 6–12 h after CPB and then decrease. The authors considered the intraoperative control of blood glucose to aid in the regulation of lactic acid levels. Ranucci *et al* (6) also reported that blood lactate levels significantly rise with the increasing duration of CPB. During heart surgery, a blood lactate concentration >3.0 mmol/l is considered to be lactic acidosis. Based on a large clinical study that included 1,376 cases, Demers *et al* (9) reported that blood lactate levels reach a peak of ≥4.0 mmol/l during CPB and that these levels correlate with the incidence of postoperative complications and mortality.

In the current study, intraoperative blood lactate levels determined during aortic cross-clamping at the aorta increased gradually with increasing operative time. Furthermore, during surgery, the levels of lactate in blood samples from the aorta reached 4.88±1.01 mmol/l and lactic acidosis was observed. Following aortic stop flow infusion and CPB, blood lactic acid levels continued to increase linearly and achieved peak levels at the end of surgery. Correlation analyses revealed that the peak levels of blood lactate were positively correlated with the duration of CPB and aortic cross-clamping. These observations indicate that blood lactate levels in non-diabetic adult patients undergoing heart valve surgery gradually increase with increasing operative time and that lactic acidosis occurs during surgery. During CPB, changes in blood lactate levels are extremely apparent and peak blood lactate levels reach high values, indicating that a number of relevant factors, other than those associated with the operation, significantly impact blood lactate levels during CPB.

Following surgery, blood lactate levels continued to rise and reached a peak of 7.66±2.33 mmol/l 6 h following admission to the ICU. The levels exhibited a downward trend towards normal ranges after 24 h, and returned to normal levels (<2.0 mmol/l) after 48 h. These findings indicate that blood lactate levels are highest 1–6 h following surgery.

Ranucci *et al* (6) observed that during CPB, high blood lactate levels are often accompanied by elevated blood glucose levels. During CPB, certain factors often lead to insulin resistance, which results in the failure of absorption and utilization of glucose by tissues. This phenomenon causes lactic acid generation through anaerobic metabolism and subsequently, lactic acid accumulation that results in lactic acidosis.

Correlation analyses in the current study revealed that during CPB, the peak blood glucose and blood lactate levels were correlated (r=0.312, P=0.009), indicating that blood glucose increases with increasing blood lactate levels. During CPB, the peak blood glucose and blood lactate levels gradually increased with increasing operative time, which was accompanied by the occurrence of hyperglycemia and lactic acidosis.

In the present study, changes in blood glucose and blood lactate levels were observed during CPB. These observations suggest that CPB-related factors significantly alter the internal environment, resulting in elevated blood glucose and blood lactate levels. Therefore, the enhancement of CPB management to improve the internal environment by controlling intraoperative blood glucose and blood lactate levels is likely to reduce the incidence of CPB-related complications and improve the prognosis of patients.

## Figures and Tables

**Table I.** Intraoperative blood glucose and blood lactate level changes (mean ± SD).

Variable	After induction of anesthesia	After clamping the aorta	After opening the aorta	Stop CPB	End of surgery
Blood glucose (mmol/l)	5.49±0.98	6.71±1.58	9.92±3.03	11.43±2.87	11.83±4.32
Blood lactate (mmol/l)	0.89±0.32	1.78±0.71	4.88±1.01	6.24±2.90	6.78±3.12

CPB, cardiopulmonary bypass.

**Table II.** Postoperative blood glucose and blood lactate level changes (mean ± SD).

Variable	Time in the ICU (h)				
	1	6	12	24	48
Blood glucose (mmol/l)	12.34±3.62	15.01±4.91	11.33±3.65	9.81±2.59	8.05±2.32
Blood lactate (mmol/l)	6.98±3.78	7.66±2.33	5.10±2.13	3.26±2.00	1.89±0.96

ICU, intensive care unit.

**Table III.** Peak glucose univariate analysis during CPB.

CPB-related factors	Correlation coefficient (r)	P-value
Lactate peak	0.312	0.009
CPB time	0.386	0.001
Aortic cross-clamping time	0.412	<0.001

CPB, cardiopulmonary bypass.

**Table IV.** Peak blood lactate univariate analysis during CPB.

CPB-related factors	Correlation coefficient (r)	P-value
Glucose peak	0.312	0.009
CPB time	0.489	<0.001
Aortic cross-clamping time	0.467	<0.001

CPB, cardiopulmonary bypass.
